# Pan African Vivax and Ovale Network (PAVON) Malaria Diagnostic Competency Training: Offering Training Opportunities to Impact Malaria Elimination Strategies in Sub-Saharan Africa

**DOI:** 10.3390/tropicalmed9120308

**Published:** 2024-12-19

**Authors:** Amidou Diarra, John Ayivase, Dolen G. Mompati, Issiaka Soulama, Mamoudou Cissé, Nancy O. Duah-Quashie, Ben Gyan, Anthony Z. Dongdem, Wisdom K. Takramah, Grace K. Ababio, Claude Oeuvray, James Mulry, Beatrice Greco, Jutta Reinhard-Rupp, Isaac K. Quaye

**Affiliations:** 1 Centre National de Recherche et de Formation sur le Paludisme (CNRFP), Ouagadougou 01 BP 2208, Burkina Faso; adoudoudiarra64@gmail.com; 2 Pan African Vivax and Ovale Network, Faculty of Computer and Allied Health Sciences, Regent University College of Science and Technology, McCarthy Hill, Accra P.O. Box DS1636, Ghana; ayivase@yahoo.com (J.A.); dolen.goitse@gmail.com (D.G.M.); soulamacnrfp@gmail.com (I.S.); cisse_m@yahoo.fr (M.C.); nquashie@ug.edu.gh (N.O.D.-Q.); bgyan@noguchi-ug.edu.gh (B.G.); dongdem@uhas.edu.gh (A.Z.D.); takramah@uhas.edu.gh (W.K.T.); gkababio@ug.edu.gh (G.K.A.); claude.oeuvray@merckgroup.com (C.O.); james.mulry@emdserono.com (J.M.); beatrice.greco@merckgroup.com (B.G.); jutta.reinhard-rupp@merckgroup.com (J.R.-R.); 3 Clinical Laboratory Unit of Institutional Care Division, Ghana Health Service Headquarters, Accra P.O. Box LG43, Ghana; 4 Botswana National Health Laboratory, Gaborone UB0061, Botswana; 5 Institut de Recherche en Sciences de la Sante (IRSS), Ouagadougou 03 BP 7192, Burkina Faso; 6 Laboratoire de Recherche, Centre MURAZ, Bobo-Dioulasso 01 BP 390, Burkina Faso; 7 Department of Epidemiology, Noguchi Memorial Institute for Medical Research, College of Health Science, University of Ghana, Legon, Accra P.O. Box LG43, Ghana; 8 Department of Immunology, Noguchi Memorial Institute for Medical Research, College of Health Science, University of Ghana, Legon, Accra P.O. Box LG43, Ghana; 9 Department of Epidemiology and Biostatistics, Fred N. Binka School of Public Health, University of Health and Allied Sciences, Ho P.O. Box 31, Ghana; 10 Department of Medical Biochemistry, University of Ghana Medical School, College of Health Sciences, University of Ghana, Korle-Bu, Accra P.O. Box LG43, Ghana; 11 Global Health Institute of Merck, Terre Bonne Building ZO, Route de Crassier 1, Eysin, 1266 Geneva, Switzerland

**Keywords:** microscopy competency, malaria diagnosis, diagnostic capacity

## Abstract

PAVON has developed a malaria microscopy competency training scheme to augment competency in malaria microscopy. Here, data accrued from training activities between 2020 and 2023 in Botswana are presented. Three trainings were done for 37 central and peripheral level technicians for a two-week period. The scheme consisted of basic theory on *Plasmodium* parasites, malaria epidemiology and diagnosis. The practicals focused on standard slide preparation, staining, parasite detection, speciation and counting. Scores were assessed by the Wilcoxon signed rank test. Participants who excelled joined the WHO External Competency Assessment for Malaria Microscopy (ECAMM). The median competency scores for the three trainings were detection: 100 (IQR = 94–100), 100 (IQR = 94–100) and 92 (IQR = 92–100), respectively, from pre-test scores of 40 (IQR = 27–54), 44 (IQR = 32–52) and 20 (IQR = 10–40) (z = 2.937, *p* < 0.003, z = 3.110, *p* = 0.002 and (z = 2.251, *p* = 0.024), respectively. Speciation: 93 (IQR = 86–96), 81 (IQR = 73–96) and 88, (IQR = 88–100) from pre-test scores of 50 (IQR = 30–50), 36 (IQR = 20–45) and 17 (IQR = 17–50) (z = 2.936, *p* < 0.003, z = 3.152, *p* = 0.002 and z = 3.237, *p* = 0.001). The competency scores achieved in the ECAMM were 98/98 and 97/98 for sensitivity and specificity pre- and post-tests detection. The training was effective in raising the competency skills of participants and is open to NMPs (National Malaria Programs) for critical capacity building.

## 1. Introduction

Developing countries in the world, and more so in Africa, carry the greatest burden of Malaria disease [1]. To ensure that this narrative changes, WHO has outlined key strategies that are to be owned by malaria endemic countries. These strategies are defined under the High Burden to High impact strategies with four key aims: (i) political will to reduce malaria deaths; (ii) strategic information to drive impact; (iii) better guidance, policies and strategies; and (iv) a coordinated national malaria response [2].

In achieving these goals, a key practical activity needed is accurate diagnosis of the parasites, to know the true burden of disease, foci of infections and how to prioritize and target these to reduce and prevent transmission and to eliminate the disease [3,4]. Generally, sub-Sharan African countries prefer the use of RDT as a routine diagnostic tool for malaria diagnosis because of the ease of use at the outpatient departments (OPDs) by nurses and technicians and by community health workers [5,6]. Confirmatory microscopy tests are done when treatments are to be initiated by an attending physician. However, for malaria elimination purposes, the use of RDT is limiting with a malaria detection threshold of 100–200 parasites/µL and unable to detect non-falciparum malaria specifically [7]. In addition, there is the problem of false negatives due to the prozone effect when excess infected blood overwhelm antigen binding sites, leading to poor binding with antibodies [7]. Similarly, false negatives are high in malaria microscopy when performed by routine microscopists whose limit of detection is between 50 and 500 parasites/µL of blood [8]. However, when done by experts, the limit of detection is significantly higher (10 parasites/µL of blood), and the data are much more reliable [9,10]. Microscopy enables differentiation of asexual from sexual parasites, parasite species and gives an insight into the transmission risks at different geographical locations [11]. These can be achieved only by experts as routinely non-highly trained microscopist would fail to report on the parasite species that are not falciparum, thereby affecting programmatic decision making [10]. Inaccurate diagnosis has the undesired outcome of unnecessary treatment when it is not required (false positives) and missed out treatment that could potentially end in fatality (in the case of false negatives) [10,12]. These scenarios are indicated in competency as sensitivity and specificity skills of malaria microscopists [13].

The need for a competent microscopy diagnostic skill set is therefore essential, as countries set their goals on elimination. Well-trained and competent malaria microscopists need to be continually available for quality assurance and to serve as support base for training new groups of competent microscopists over time for malaria disease surveillance [14]. Several countries have reported on the positive impact of malaria microscopy training for staff of the Ministry of Health and, specifically, the National Health laboratories (NHLs)/National Malaria Programs (NMPs). These included improved malaria epidemiological knowledge and competency in detection speciation and counting [15,16].

PAVON has one of its goals as training of competent microscopists using both theory and practice as the mode of teaching. The intent is to assist NMPs/NHLs to build the critical capacity for surveillance towards elimination. Here, we report on the impact of such training conducted in Botswana in collaboration with the National Health Laboratory and the National Malaria Program to provide personnel support for malaria elimination.

## 2. Materials and Methods

### 2.1. Training Design and Content

The training was prospective and included participants who worked in various health facilities across the country as laboratory technicians, technologists or laboratory assistants. Some of the trainees had limited training on malaria microscopy and limited access to microscopes at their facilities. This prompted PAVON to initiate a program of donating microscopes as part of the training package. These donations were facilitated by Rotary Against Malaria-Global group (RAM-G) and Merck Global Health Institute, Geneva.

#### 2.1.1. Training Site and Participants

Selection of the training site and participants was at the discretion of the Ministry of Health (MoH) and or National Health Laboratory (NHL). For the site, training was usually at a location away from distractions and close to a relatively well set up laboratory with space for slide preparation, buffer solution preparation, staining and drying. For the participants, the MoH/NHL took cognisance of district representation, the role of the participant in the laboratory at the district, gender balance and potential to learn new skills and/or improve on malaria microscopy skills. Each training session enrolled between 11 and 14 participants.

#### 2.1.2. Training Approach

Each training session was residential and spanned two weeks. The scheme used the WHO guidelines and modified it to suit the local context and needs. This was with regards to the theoretical knowledge in malaria disease, skills and exposure to practical microscopy at the laboratories where they normally worked. All trainees held either biomedical science certificate or degree or medical laboratory certificate or degree.

#### 2.1.3. Training Facilitators

Facilitators were a combination of WHO certified Level 1 malaria microscopy experts (3) who also serve as national malaria microscopy and National Health Laboratory supervisors in their respective countries and established malaria researchers (5).

#### 2.1.4. Training Content

The theoretical training materials covered the following topics: basic epidemiology, malaria epidemiology, parasite life cycle, key metrics for malaria microscopy and definitions of key terminologies in epidemiology and microscopy. There was also a pre-test theory that focused on parasite detection, stage of development and speciation. For the practical, the content covered slide preparation, staining and viewing under the microscope for parasite detection, stage of development, speciation and counting.

### 2.2. Basic Guidelines on Slide Preparation

**Figure 1:**
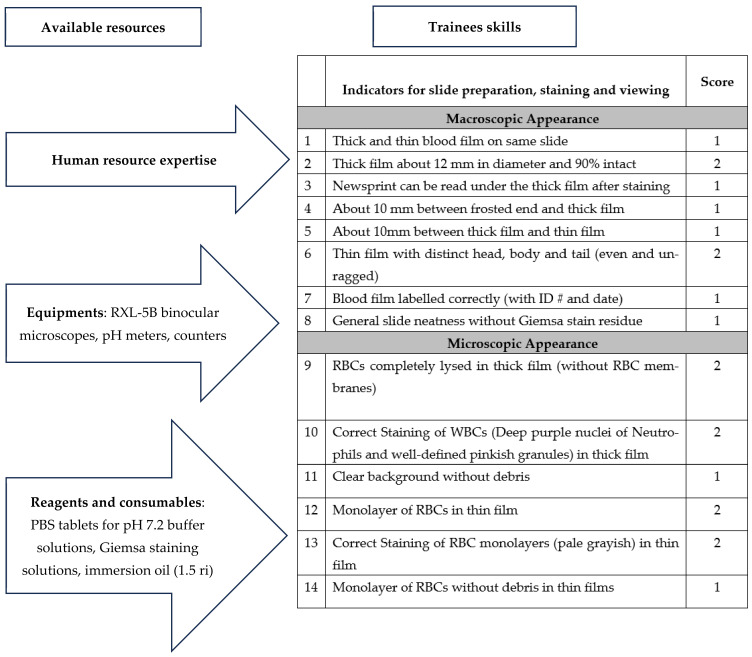
Scheme for basic guidelines on slide preparation and scores for competency.

The methods were consistent with the WHO recommendations already available in the guidelines, manuals and standard operating procedures for the preparation and staining of blood films that are of high quality [13,14] (Figure 1). Additional tips based on the experiences of the facilitators were shared, which included what to do if basic consumables and tools are unavailable in laboratories. Some of the recommendations done in Sri Lanka, which had been tested under those circumstances and found practicable, were alluded to [13,14,17]. These were (a) to wipe new slides clean with a tissue before use for blood smears, (b) thick and thin blood smears were to be prepared on the same slides but with the necessary precaution to ensure a gap of about 1 cm between the two smears to prevent fusion and methanol flow from the thin film into the thick film, (c) labelling of the slides on the side facing away from the thin film to prevent wash-offs and (d) collection of blood for the thick smear first before that of the thin smear, while keeping the standard preparation of the thin film first and then the thick film. For drying, the films were prepared just before the lunch break so that, by the time lunch was over in approximately 135 min, the slides were dried enough for staining. In the absence of smooth-edged slides for spreading, participants were told of alternate methods, including the use of sandpapers, to smoothen the edges of the slides.

### 2.3. Criteria for Defining the Quality of Blood Smears

**Figure 2:**
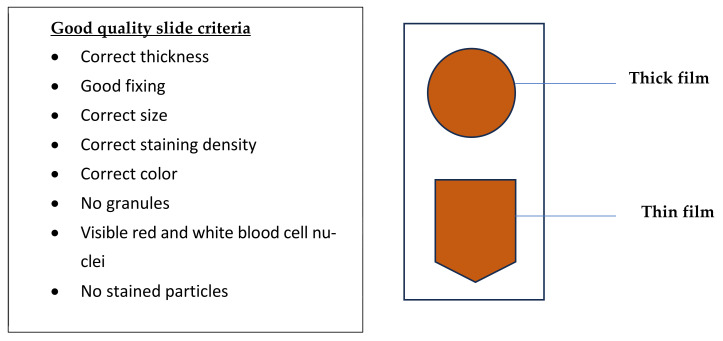
Criteria for defining the quality of the blood smears.

Following the guidelines of WHO for quality of the slides, the blood films were designated as either good or bad (Figure 2). A good film was defined as having the following characteristics: correct thickness, good fixing, correct size, correct staining density, colour, absence of staining granules, red, white blood cell nuclei and absence of stained particles. Specifically, a thick smear that has the correct thickness and is well fixed should allow for (a) the examination of a hundred microscope fields with a WBC density of 5- > 10 per field. (b) it is of the correct size if it is sufficient to cover 100 fields. (c) If WBC nuclei are red or purple, then staining is of the correct degree; (d) where staining granules are unable to obscure the presence of parasites, they are said to be low. If any of the above was lacking, the smear was deemed as ‘bad’. Similarly, a thin smear was adjudged of good quality if (a) two hundred microscope fields with a density of nearly 250 RBCs could be examined, (b) staining makes WBC nuclei red or purple and (c) stain granules and particles are low to the extent that they do not obscure parasite identification. If any of the above was lacking, the thin smear was considered as of ‘bad quality’ [13,14].

### 2.4. Set of Training Slides

The slide set used in the training was exactly as specified in the ECAMM training slide set, which is presented in Table 1. These were 178 slides of carefully prepared and Giemsa-stained blood films that were either malaria-positive with varying parasite densities or malaria-negative. The slides covered all the four common *Plasmodium* parasites known to be infectious to humans (*P. falciparum*, *P. vivax*, *P. malariae* and *P. ovale*). There were additional negative slides of *Plasmodium* parasites that included other parasites (*Babesia* spp., *Trypanosoma* spp. and *Microfilariae*) that could cause symptomatic infections as *Plasmodium* species and may be mistaken for debris. The slides were obtained from the WHO Collaborating Centre for malaria diagnosis, Western Pacific Region at the Research Institute for Tropical Medicine (RITM), Muntinlupa City, the Philippines. The slides are designed to assess malaria microscopists’ competence in parasite detection, species identification and quantitation at the level of a patient.

#### 2.4.1. Combination of Slides Used for Detection

The number of slides used in the detection training were 20 negative slides and 20 positive slides all within the range of low to medium density p/µL of blood as specified in the slide types.

#### 2.4.2. Combination of Slides Used for Species Identification

The same number and set of slides used for detection were used for species identification.

#### 2.4.3. Combination of Slides Used for Quantitation

The slides used for quantitation were only *P. falciparum* from ≥200 p/µL to ≤2000 p/µL.

#### 2.4.4. Time Allocated for Each Slide Reading

Ten minutes was allocated to each slide reading irrespective of the category: detection and developmental stage, speciation and quantitation. In counting, if parasitaemia was estimated to within 20% of the approved range, it was deemed accurate.

### 2.5. Statistical Analysis

All the results were entered into a Microsoft excel file. Descriptive statistics (frequencies and percentages) were used to calculate categorical variables. For statistical analysis, the Vassar statistics website tools were used (www.vassarstats.net) (accessed on 3 December 2024). The median and interquartile ranges were used as measures of central tendency to estimate the overall scores. The Wilcoxon matched pairs signed rank test were used to compare median scores pre- and post-tests. The skills set of each participant was determined by calculating the sensitivity and specificity of the detection skills.

## 3. Results

The number of participants for the first, second and third trainings were 11 (3 males, 8 females), 12 (4 males, 9 females) and 14 (7 males, 7 females) respectively. All the participants from the first training had undergone previous online theoretical training. However, none of the participants in the subsequent two trainings had undergone a previous malaria microscopy training anywhere. In the first training, eight were from government hospital laboratories, one from the National Health Laboratory, one from the malaria program and one from the Botswana Quality Assurance Laboratory. In the second training, nine were from government hospital laboratories and three from clinics. In the last training, 10 were from government hospital laboratories, 1 from the National Blood Transfusion Centre and 3 from clinics.

### 3.1. Writing Theory

**Figure 3:**
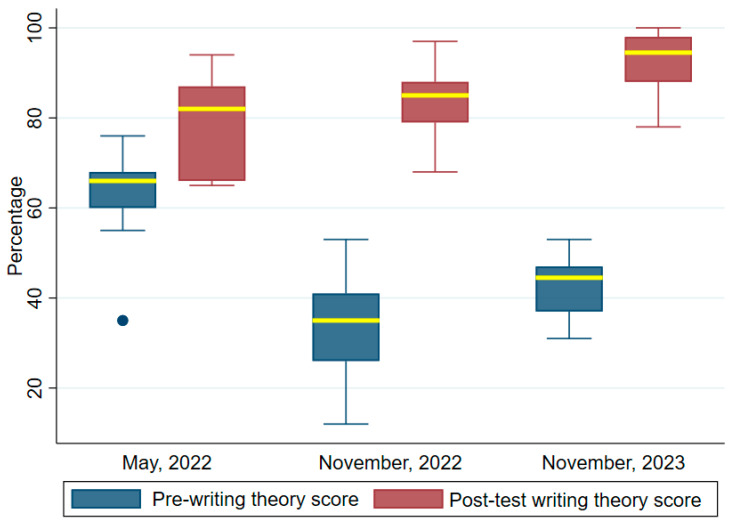
Comparing writing theory test scores before and after training. The median values are highlighted in yellow bounded by the upper and lower quartiles. A filled dot is an outlier.

Figure 3 shows the test scores pre- and post-test for the writing theory. The median pre- and post-test scores for the first, second and third trainings were, respectively, pre-test 66 (IQR = 60, 68), post-test 82, IQR = 66–87)}, (pre-test, 35, IQR = 36–41), post-test (85, IQR = 79–88)} and pre-test 45.5 (IQR = 37–47) and post-test 94.5 (IQR = 88–98). The Wilcoxon signed rank test was conducted to ascertain statistical significance between the median writing theory test scores pre- and post-training. The differences were statistically significant. The z-scores and *p*-values were first (z = 2.852, *p* < 0.004), second (z = 3.184, *p* = 0.002) and third (z = 3.301, *p* = 0.001), respectively. These results indicated that the training program significantly improved the knowledge of the participants in basic malaria epidemiology, life cycle and disease presentation.

### 3.2. Identification (Detection)

**Figure 4:**
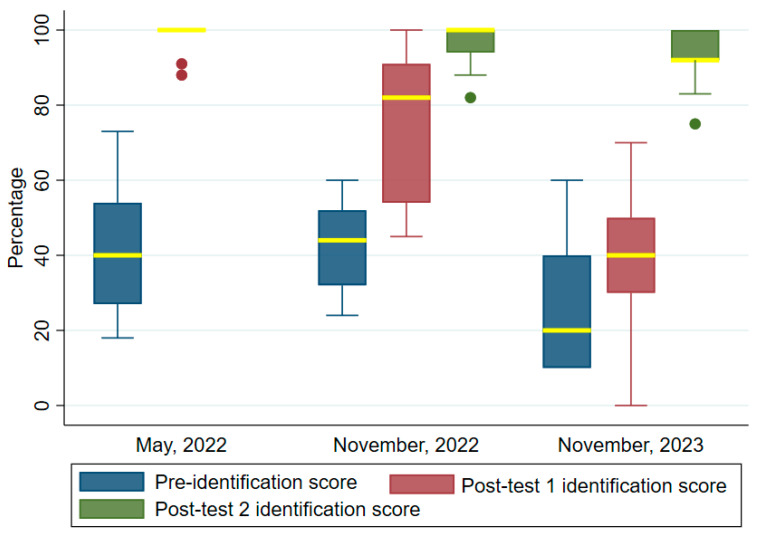
Comparing identification test scores before and after the trainings. Two post-tests were conducted in the November 2022 and November 2023 trainings. The median values are highlighted in yellow bounded by the upper and lower quartiles. Filled dots are outliers.

Figure 4 shows the median scores in the identification of the parasite before and after the training programs. Notably, the median scores recorded after the training were higher than that before the training. For the first training, the median scores were pre-test: 40 (IQR = 27, 54) and post-test: 100 (IQR = 94–100). Two post-tests were conducted in the second (November 2022) and third (November 2023) trainings. The median scores were, respectively, pre-test: 44 (IQR = 32–52), post-test1: 82 (IQR = 54–91) and post-test 2: 100 (IQR = 94, 100). In the third training, the pre-test median score was 20, IQR = 10, 40), while the post-tests 1 and 2 were 40 (IQR = 30–50) and 92 (IQR = 92–100). Wilcoxon signed ranked tests showed that the increases in the post-tests in all three trainings were highly significant: (z = 2.937, *p* < 0.003, z = 3.110, *p* = 0.002 and (z = 2.251, *p* = 0.024), respectively.

### 3.3. Speciation

**Figure 5:**
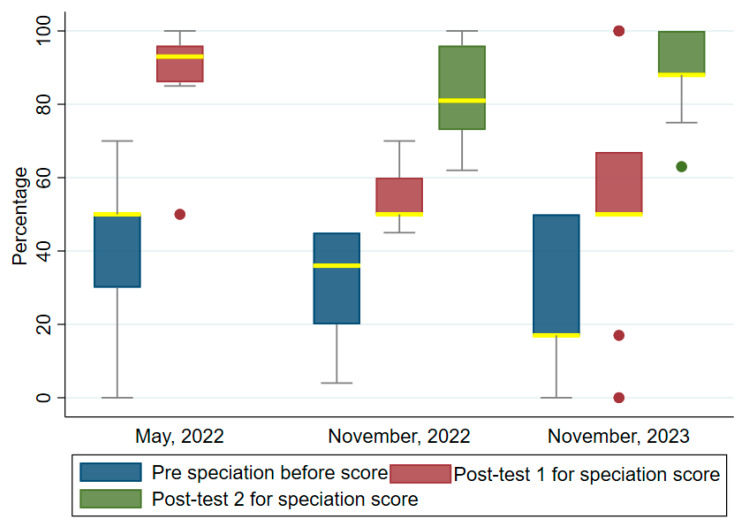
Comparing the speciation test scores before and after the trainings. Two post-tests were conducted in the November 2022 and November 2023 trainings. The median values are highlighted in yellow bounded by the upper and lower quartiles. Filled dots are outliers.

Figure 5 shows the differences in the pre- and post-test median scores for speciation for the first, second and third trainings. Like the previous performances, the post-test median scores were higher than the pre-test median scores. These were, respectively, pre-test: 50 (IQR = 30–50) and post-test: 93 (IQR = 86–96). The second training pre- and post-tests 1 and 2 median scores were, respectively: 36 (IQR = 20–45) 50 (IQR = 50–60) and 81 (IQR = 73, 96). The third training pre- and post-test median scores were, respectively: 17 (IQR = 17, 50), 50 (IQR = 50–67) and 88 (IQR = 88–100). The Wilcoxon signed ranked test indicated that the increases were highly significant: first training (z = 2.936, *p* < 0.003), second training (z = 3.152, *p* = 0.002) and third training (z = 3.237, *p* = 0.001).

### 3.4. Counting

**Figure 6:**
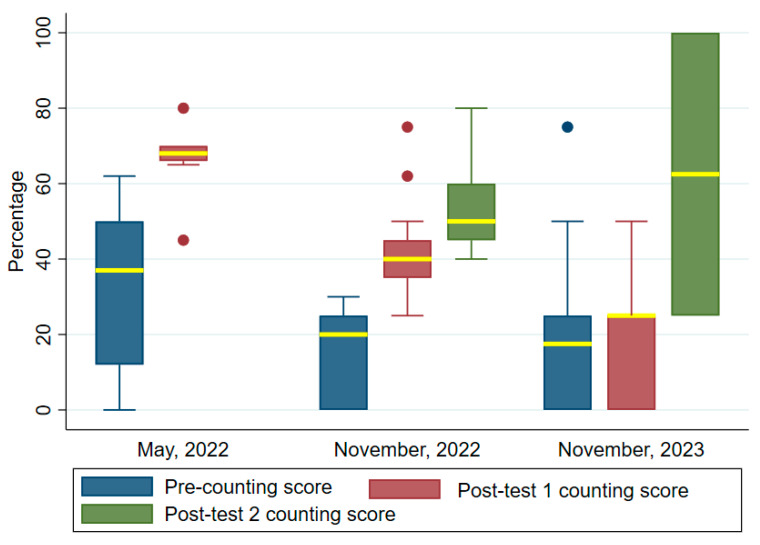
Comparing counting test scores before and after the trainings. Two post-tests were conducted in the November 2022 and November 2023 trainings. The median values are highlighted in yellow bounded by the upper and lower quartiles. Filled dots are outliers.

Figure 6 shows the differences in the median scores for counting pre- and post-training. Quantification of the parasites were done only with *Plasmodium falciparum*-positive slides. The median scores increased post-training, as in the previous skills set. The specific values were first training pre- and post-test: 37 (IQR = 12–50) and 68 (IQR = 66–70). The second training pre-and post-tests 1 and 2: 20 (IQR = 0, 25) and test 1 50 (IQR = 35–45) and test 2 50 (IQR = 45–60). In the third training, the pre-and post-tests 1 and 2 scores were: 7.5 (IQR = 0, 25) and 25 (IQR = 0–25) and 62.5 (IQR = 25–100). The increases in counting skills were highly significant for the first and second trainings but not the third. The Wilcoxon signed rank tests, respectively, were z = 2.94, *p* = 0.003, z = 3.114, *p* = 0.002 and z = 0.662, *p* = 0.508.

### 3.5. Mixed Infection

**Figure 7:**
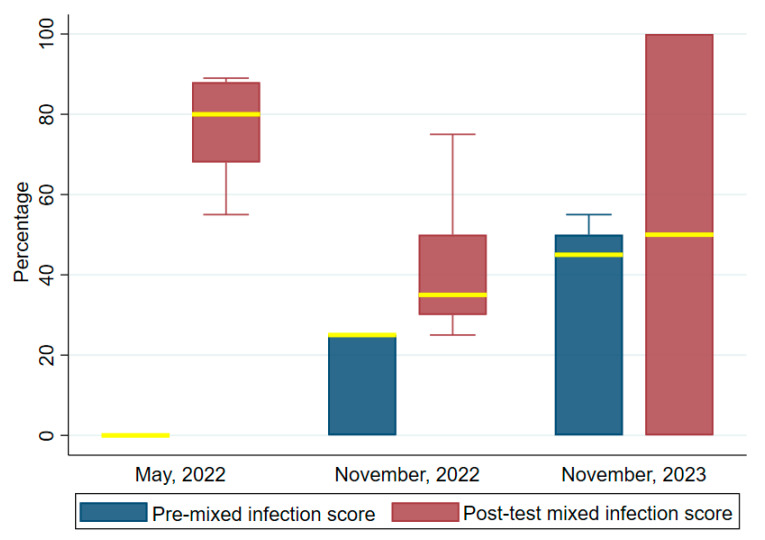
Comparing mixed infection detection test scores before and after the trainings. The median values are highlighted in yellow bounded by the upper and lower quartiles.

Figure 7 shows the differences in the median scores for mixed infection detection before and after the trainings. The median scores recorded after the post-tests were significantly higher than before the trainings, as observed in the other skills set. The first training median pre-and post-test scores were, respectively: 0 and 80 (IQR = 68–88). For the second and third trainings, the scores for the pre- and post-tests were, respectively: 25 (IQR = 0–25) and 35 (IQR = 30; then 45 (IQR = 0–50) and 50 (IQR = 0–100). Wilcoxon signed ranked tests for the increases in scores regarding competency in the first, second and third trainings were highly significant: z = 2.936, *p* = 0.003, z = 3.089, *p* = 0.002 and z = 2.618, *p* = 0.008.

### 3.6. Sensitivity and Specificity of the Participants Skills in Detection and Speciation

The overall skills set of the participants in how they were able to avoid false positive (sensitivity (%)) and false negatives (specificity (%) were 98/97, 94/88 and 93/82 for the three trainings, respectively. Participants from the first two groups who excelled and were selected to undergo the ECAMM training had median sensitivity and specificity of 98/98 and 97/98 for pre-and post-tests, respectively. The training competencies acquired through the training delivered by PAVON were equivalent to those obtained through the ECAMM training.

## 4. Discussion

The present report provides evidence of the importance of malaria microscopy training to equip NMPs with the critical mass of competent staff who can be used in the malaria elimination agenda at all levels. The findings align with other trainings previously reported in sub-Saharan Africa [18,19,20,21]. Counting and detection of mixed infections were the most difficult for the trainees. This was not unique, which is why the WHO training competency assessment cutoff mark for counting and mixed infection is at >50% compared to detection and speciation, which are at >90% for level 1 and 80% for level 2 [13]. In terms of specificity and sensitivity, the suggested thresholds are 97 and 90%, respectively, for the highest competency level [13,22]. The main emphasis in clinical disease management of malaria is whether a slide is positive or negative for malaria to trigger treatment. As a result, technicians in sub-Saharan Africa focus more on detection and, in most cases, detection of *P. falciparum* and less on other species. It is clear from our training that a significant increase in the competency level of technicians can be achieved if training is sustained. This will also impact on their ability to speciate correctly and efficiently. Outside of limited training opportunities, the sheer volume of work and poor supply of quality reagents could adversely reduce competency skills retention.

African Leaders Malaria Alliance (ALMA) have accented to the goal of malaria elimination, giving impetus to National Malaria Programs (NMPs) to see that malaria elimination is attainable [22,23,24]. Such support is warranted, as are collaborative activities between malaria focused research networks and NMPs to provide the skills set and competency for accurate data acquisition. Microscopy remains an important tool in malaria disease surveillance, case management and anti-malarial drug efficacy and resistance [25]. We found that participants who had gone through a previous online training (first training group) achieved higher competency scores than those who had not (second and third training groups). Nevertheless, an intensive hands-on training can help to improve those who are less exposed to additional trainings. This has also been seen in other training programs [19,25,26]. Here, we emphasise that, although video clips of malaria microscopy training are useful, repeated hands-on trainings are crucial for the acquisition and sustenance of competency skills. By providing good microscopes as a component of our training, we ensure that the programs have at least the minimum tools they can use to sustain further training.

It was notable that the participants who had attained level A by our grading system achieved level 1 status in the ECAMM competency assessment, with similar sensitivity and specificity outcomes. This is remarkable and shows that malaria research networks that have established excellent training schemes can play vital roles in capacity building in malaria microscopy. Such networks could be recognized by the WHO and RBM to augment capacity training in malaria competency. In this way, NPMs will not be limited in their malaria microscopy capacity building agenda. Currently, only AMREF and the University of Cheikh Anta Diop de Dakar (UCAD) are recognised by the WHO for the ECAMM. We think that this is woefully limiting and needs to be expanded.

## 5. Conclusions

We conclude that, to build a reference core of highly skilled microscopists to sustain competency skills training for NMPs, continued training would be necessary. This training must be validated by competency level awards so both the NMPs and trainees know the competency skills of the staff and how they can be utilised in the malaria elimination agenda. We have shown here that institutions with such training capacity can significantly augment the availability of highly competent malaria microscopist for programs.

## Figures and Tables

**Table 1:** Composition of the slides set used in the training.

Malaria Species	Parasite Density/µL	Number of Slides
Negative slide	0	20
*Pf* low density	80–200	5
*Pf* low density	200–500	5
*Pf* medium density	500–2000	5
*Pv* low density	80–200	5
*Pv* low density	200–500	10
*Pv* medium density	500–2000	10
*Pm* low density	80–200	10
*Pm* low density	200–500	10
*Pm* medium density	500–2000	10
*Po* low density	80–200	10
*Po* low density	200–500	10
*Po* medium density	500–2000	5
Mixed inf (*Pf* + *Pm*)	positive	10
Mixed inf (*Pf* + *Po*)	positive	10
Mixed inf (*Pf* + *Pv*)	positive	10
Mixed inf (*Po* + *Pm*)	positive	10
Mixed inf (*Po* + *Pv*)	positive	10
Mixed inf (*Pv* + *Po*)	positive	10
Other blood parasites (*Babesia* spp., *Trypanosoma* spp., *Microfilariae*)	Positive	3

## Data Availability

The raw data supporting the conclusions of this article will be made available by the authors on reasonable request.
